# Abstracts and Gleanings

**Published:** 1884-06-20

**Authors:** 


### ABSTRACTS AND GLEANINGS.

**Toothache.**—Dr. J. R. Irwin in the North Carolina Medical Journal says: A great many remedies have been recommended for this common and very painful affection. One of the best and most pleasant things that can be used to relieve this painful state of the dental nerves, is chewing cinnamon bark. It destroys the sensibility of the nerves and suspends the pain immediately, if the bark is of good quality. After repeated trials, and in different cases, I am convinced that it is generally as efficacious as any of the other remedies, suggested for odontalgia, and not attended with the unpleasant consequences of creosote, carbolic acid, &c., which relieve the pain, but leave the mouth as sore and painful as the tooth was previously, though these results are usually due to carelessness in using.

**Apomorphia in Infantile Convulsions.**—Dr. Edward Cottrell, in the Medical Press and Circular, reports the following:

On November 11, 1883, I received a summons requesting my immediate presence to a child in a fit. Upon my arrival I found the patient, a child, aged eighteen months, suffering from typical infantile convulsions. The mother stated that the child was attacked about half an hour after its dinner, which upon inquiry I found consisted principally of greens and potatoes. The attack was preceded by vomiting. There was a great congestion of the veins of the neck, and the breathing was stertorous. I immediately proceeded to use artificial respiration by the Marshall Hall method, and after about five minutes the breathing became less stertorous, and the cyanosis less. I thought it would be a good plan to evacuate the contents of the stomach, in spite of the mother's assurance that the child had returned all its dinner, and, failing to produce reflex vomiting by irritation of the fauces, I procured some apomorphia. I administered two minims of a two-per-cent. solution of this drug subcutaneously, and in one hundred seconds the stomoch evacuated its contents—a prodigous quantity—with hardly any effort.

Immediately after this the convulsions ceased, and the child became quite conscious, nor has it subsequently had any attacks of a like nature.—*N. C. Med. Journal.*

**The Sixth Cholera Report of Dr. Koch.**—Following very closely after Dr. Koch's fifth report, dated January 8th, comes another, and probably the last one for the present, dated February 2d, at Calcutta.

This latest bulletin from the cholera commission is both interesting and disappointing; interesting because Dr. Koch now announces definitely and positively that he has discovered the cholera-bacillus, but disappointing because he does not give us the demonstrative evidence that his announcement is true.

The organisms which the commission believe to be the cause of cholera are described as follows:

"They are not quite straight, as other bacilli are, but slightly curved, comma-like. The curvature may be even so marked that the stave may almost present a semi-circular form. From these curved staves, S-shaped figures, and more or less elongated, slightly undulating, linear forms may be developed in 'pure cultivations,' of which the first two segments and the last correspond to the form of the bacilli as found in cholera, and which, continuously increasing in number, remain attached to one another." Furthermore, they manifest very lively voluntary movements, which are best seen when examined in a drop of nutritive fluid suspended from the under surface of a cover-glass. In such a preparation the bacilli may be seen to swim with the greatest celerity across the field. Their behavior in nutrient gelatine is particularly characteristic; they form colorless colonies in it, in which are closed at first, and look like strongly refringent accumulations of glass-fragments. These colonies gradually liquefy the gelatine, and extend themselves over a limited area. Further, they may be distinguished with tolerable certainty when cultivated in hollowed slides, as they are always found at the margin of the drop of nutritive fluid, and their peculiar movements may be observed as well as their comma-shaped forms after the application of solutions of anilin-dyes.

These bacilli were found in all the intestinal discharges of cholera-cases, and in the contents of the intestinal canal at post-mortem examinations, but not in any other disease, nor were they to be found in specimens of dirty water from various sources. As it is known that cholera-like symptoms are induced by arsenic, an animal was poisoned with this substance, but cholera-bacilli could not be detected.

They were only twice observed in the vomit. In the early feculent dejections only a few were present. As the dejections became odorless and watery they appeared in great numbers, while all the other bacterial forms coincidently disappeared. As the dejections returned to a natural character again, the bacilli disappeared.

Inoculation of the cultivated bacilli in lower animals uniformly failed to produce the disease. Dr. Koch believes, furthermore, that such attempts will always fail, because although cholera has been endemic throughout Bengal for years, no instance of the disease occurring in animals is known.

The opinion that the peculiar bacillus described is the specific cause of cholera rests upon other evidence, therefore, than that of inoculation. This evidence consists mainly in the fact that the organism is limited to the seat of the disease, that it is not found in other tissues or in other diseases, and that its activity keeps parallel with that of the cholera process. To the very obvious objections that the growth of the peculiar bacillus is simply favored by the peculiar conditions of the disease, Dr. Koch gives answers which cannot possibly satisfy scientific criticism. The great confidence felt in the carefulness and skill of the distinguished investigator, however, will lead many to accept his judgment, but such acceptance must as yet be based more on faith than sight. In fine

Dr. Koch has found a bacillus, but he has not found, for science, the cholera-bacillus.—*N. Y. Med. Record.*

**Canned Foods.**—There is deemed to be no good cause for the cry which has been raised against foods put up in tin cans, except in the acid preparations, which may corrode and lead to the development of poisonous elements. Professor Attfield, after a careful investigation, says:

"In my opinion, given after well weighing all evidence hitherto forthcoming, the public have not the faintest cause for alarm respecting the occurrence of tin, lead, or any other metal in canned foods."

**A Caution About Jequirity.**—After reporting a case of sloughing of the cornea after the use of jequirity, in the Weekly Medical Review, February 13, 1884, Dr. S. Pollak formulates as follows:

1. Jequirity is by far the best remedy which has been hitherto used for trachoma and pannus.
2. It does all, and more speedily, that has ever been claimed for purulent inoculation, minus the repulsiveness of the last remedy.
3. The infusion of jequirity must be used only when perfectly fresh. After four or five days it swarms with bacteria, when the danger of their entering the tissue and causing a septic state is very great.
4. Sterilizing the infusion requires much care and labor, and may not always be practicable. It will doubtless retard the decomposition, but it will not prevent it entirely.
5. The full therapeutic utility of jequirity will only be attained when chemistry shall have succeeded in preparing an alkaloid of it, which will keep, and the strength of it is properly known.—*Med. and Surg. Rep.*, March 22.

**Turpentine as a Prophylactic in Infectious Diseases.**—The Medical Record tells us that H. Vilandt writes in the Ugeskrift for Leager, vol. viii, No. 8, 1883, concerning the value of the oil of turpentine in the treatment and prophylaxis of diphtheria and the exanthematous diseases. He states that he has never seen any of these diseases spread from a sick child to other members of the family when this remedy was employed. In many of his cases no isolation could be attempted, as the mother was the only female in the family, and was obliged to take care of both the sick and the well, continually passing back and forth from one to the other. His method was to pour from twenty to forty drops of a mixture of equal parts of turpentine and carbolic acid into a kettle of water, which was kept simmering over a slow fire, so that the air of the sick-room was kept constantly impregnated with the odor of these two substances. He claims also that by this means a favorable influence is exerted upon the exudation in diphtheria, although it is by no means curative of the disease, and should never be relied upon to the exclusion of other remedies.—*Med and Surg. Rep.*, March 29, 1884.

**Surgical Errors.**—Dr. John B. Roberts, in Pennsylvania State Medical Society (Med. News), said:

Many surgical theories and procedures have become traditional, and are accepted as true and correct, merely because reverence for antiquity or careless acceptance has not questioned their right to be classed as surgical facts. The present age is an incredulous one, and demands accurate investigation of all such claims. The field of investigation is large, for progress has been retarded by the influence of theorizing writers with mono-chromatic vision, the example of non-seeing and non-looking devotees of the fetiches of surgical superstition, and the convincing effect of a repetition of false statements. The author selected a few topics which have greatly interested him, and concerning which he probably differed quite widely from many.

Adherence to chloroform in the face of well-known facts as to its danger is criminal when circumstances permit ether to be obtained. The assertion that it is often impossible to produce anæsthesia with ether is the result of inefficient methods of administration. Ether, if given as chloroform is and should be given, is a useless anæsthetic; but, given properly, it is efficient.

VALUE OF STYPTICS.—The belief in the necessity of styptics is a delusion less dangerous, but is given more extended credence. Such agents are seldom, probably never, needed in general surgery to arrest hemorrhage. When ligatures, torsion, or acupressure is not demanded (and such is seldom the case unless the artery is as large as the facial), moderate direct pressure applied in dressing the wound is the only hemostatic required. Styptics often do harm; and, as they are not needed, they should be discarded.

DANGER OF TREPHINING THE SKULL.—The dislike to make exploratory incisions in closed fractures of the skull evinced by some surgeons, and the objection of others to trephining and thus opening the diploic structure in open fractures, are delusions of a most disastrous tendency. To wait until symptoms of a cerebral compression or inflammation have supervened is to lose the most favorable opportunity for mechanical relief. Such a Fabian policy is often followed by death. The treatment of open and of closed fractures of the skull should not be looked upon as very different, since, with the present improved methods of dressing wounds, the successful issue depends almost entirely upon the cerebral rather than the cranial phase of the injury. If such fractures as are usually seen in the skull were not in proximity to the brain, the surgeon would consider them almost trivial. The feature of closed fractures that renders them so troublesome is the obscurity that accompanies them. The author has for a number of years strongly advocated making closed fractures open ones by means of an exploratory incision whenever there is a suspicion of the existence of depression or splintering. In open fractures, operations to elevate depressed portions, and to get rid of splinters of the inner table thrust into the membranes, should be undertaken. It is better to err on the side of action than that of inaction. Careful manipulation and proper dressings at an early stage are sources of less risk than is incurred by the surgeon who leaves unseen and unsuspected fragments thrust into the membranes or brain.

OPERATIVE DELAY IN STRANGULATED HERNIA.—A similar delusion of fatal issue is that leading to postponement of operative interference in strangulated hernia. Repeated attempts at forcible taxis and medical pow-wowing with temporizing measures, have ended more lives than the use of the knife. Herniotomy, done within twelve hours, is almost always followed by recovery. Death is to be expected, however, if strangulation has existed for two or three days; and the gut has been bruised by violent manipulation in the endeavor to relieve the constriction by taxis. Moderate taxis under ether, a half day's treatment with cold applications and the internal use of morphia, and a second moderate attempt at taxis, followed, if unsuccessful, by immediate operation, is the sequence to be followed in strangulated hernia. When symptoms of strangulated hernia exist, the slightest fullness and tenderness in one groin over either of the rings, is a sufficient localizing indication to warrant operation.

OPERATIVE DELAY IN ACUTE PHLEGMONOUS INFLAMMATION.—No insane delusion, no Spanish Inquisitor, ever caused so many hours of excruciating physical torture as the hallucination that acute abscesses and furuncles must not be incised until pointing has occurred. All the world knows that evacuation of imprisoned pus in phlegmonous inflammation means instant relief of the agonizing pain; yet how few of the profession early and freely incise such inflamed tissues unless they first see the yellow pus under the thinned skin or feel the fluctuation of the fluid in the abscess cavity. The pain is caused by the effort of the pus and the sloughing tissue to escape. Is it not, then, more rational to make a free incision to-day than to wait till next week? Time and pain are both saved by early incision. If the cut is made before the pus is formed, so much the better. Probably no form of abscess needs early and free incision more imperatively than that under the palmer fascia. Destructive burrowing of pus is prevented by this radical procedure; which also saves the patient many days of poultices and purgatory.

OPERATIVE DELAY IN MALIGNANT TUMORS.—Much bad surgery results from a delusive postponement of operative interference in malignant diseases. Instant removal is to be practiced in such cases, provided the patient is deemed fit to stand the surgical shock.

NECESSARY FATALITY OF TRAUMATIC TETANUS.—That traumatic tetanus is, of necessity, fatal, is a commonly held opinion. Proper treatment is sometimes neglected because of this belief in its hopelessness. That the prognosis is extremely unfavorable was admitted, but that cases of a severe type recover is undoubted. Chloral hydrate in full doses has given the best results; but he merely wished to impress the fact that a fair number of cases of traumatic tetanus recover.

FATALITY OF PERICARDIAL AND CARDIAC WOUNDS.—The prevalent notion of the excessive danger of these wounds is delusional; at least in as far as it teaches that these structures will not brook surgical interference. The pericardial sac should be dealt with exactly as the pleural sac by aspiration, incision, irrigation, and drainage, according to the lesion. That simple puncture or aspiration of the heart itself is not accompanied by the expected risk to life has been pretty well shown, though I am not prepared to recommend its general adoption for trivial cardiac conditions.

**The German Cholera Commission.**—The German Cholera Commission, Drs. Koch, Gaffky, and Fischer, returned to Berlin from Calcutta on May 3d. Dr. Ewald, in an editorial announcement of their arrival, in the Berliner Klinische Wochenschritt of May 12th, says:

"It is seldom that a scientific expedition is conducted with so great moderation in every respect, and for every interest, as this, upon which the eyes of the nation, from the Emperor down to the most obscure man, have been directed with active interest."

The Commission has been received with great honors, but the honors of the Government have not been empty. In the Reichstag, Herr von Botticher brought forward a bill to place the sum of 135,000 marks (over $30,000) at the disposal of the Emperor, in order to reward the members of the German Cholera Commission for their brilliant and important discoveries in Egypt and India. Herr von Botticher, in proposing the bestowal of the honorarium, spoke in very graceful terms of the services of this Commission: "It was entirely owing to Dr. Koch's readiness to place himself at their disposal that the Government was able to send out the Commission so quickly." The bill was passed, on May 13th, by acclamation, when Professor Virchow welcomed this, the first occasion, as he said, on which he had ever been able to give his full support to the Government, and paid a high compliment to the energy, the diligence, the intelligence, and the disregard of danger, which had won for German science such a splendid victory. But, though Dr. Koch had discovered the bacillus or germ of cholera, medical men should not be too sanguine about the future prevention and cure of the disease.

Of the sum, 100,000 marks will be assigned to Dr. Koch, the chief of the Commission, and the rest divided among his colleagues. A less substantial, though equally honorable, reward has been conferred on Dr. Koch, in the shape of a high decoration—the Crown Order of the second class, with the star; and Fischer and Gaffky with the Order of Red Eagle. Lastly, at a sitting the other day of the Imperial Board of Health, the great nosological explorer, in addition to being officially eulogized for his successful researches, was presented with a life-size bust of the Emperor, by a distinguished German sculptor.—*Med. News.*

**A Liniment for Rheumatism.**—The Quarterly Therapeutic Review says, methyl salicylate (oil of wintergreen) mixed with an equal quantity of olive oil or linimentum saponis, applied externally to inflamed joints affected by acute rheumatism, affords instant relief, and, having a pleasant odor, its use is very agreeable.—*Med. Digest.*

**Boracic Acid.**—Dr. Squibb writes of boracic acid or, more properly, according to the nomenclature of the late Pharmacopœia, boric acid, as follows: "If the powder be needed, as is generally the case, it should be specified in the prescription. The powder should be very fine, and should be white and light, and entirely free from particles when rubbed between the finger and thumb, feeling very like powdered soap. It is only such powder that answers well in eye surgery or general surgery for dressings, and solutions are also best made from it. A saturated solution contains about nineteen grains to the ounce, and from ten grains in the ounce to saturation it is used as an eye-wash or to granulating and suppurating surfaces. It is a very bland and soothing application, both in powder or solution, relieving irritation and arresting suppuration. It is a potent antiseptic, much less expensive than salicylic acid, and is odorless and more easily managed than carbolic acid. It is probably better than either to preserve hypodermatic solutions. In surgical dressings it has the great advantage over carbolic acid of not being irritant nor poisonous. But not being volatile it does not deodorize the air."—*Ephemeris.*

**Rupprecht on the Treatment of Boils and Carbuncles.**—Dr. Rupprecht, of Hettstadt (Deutsche Med. Wochensch.), regards feruncles, carbuncles and anthrax pustules to be all dependent on an infectious cause, and the same treatment to be suitable for all of them. In boils, he removes the little scab which always forms early on the top, and presses into the purulent cavity a little piece of cotton wool moistened with spirit of ammonia. This ought to be done six or eight times at a sitting, a fresh piece of wool being used each time, and it may be necessary to repeat the treatment on the following day. In very large boils scarification, and in carbuncles a cross incision, must precede the application of the ammonia; in anthrax the scab must be removed and the surrounding tissue scarified in a radiating form. The parts should be dressed with boracic ointment after cauterising, and it generally heals without causing any disturbance. Boils in the external ear, where septic material is easily conveyed by the fingers, should be incised with a small knife and then dressed with some antiseptic which will not injure the tympanum, such as thymol, boracic acid, or iodoform.—*Lond. Med. Record.*

**Quebracho in Dyspnœa.**—El Sentido Captol, en las Ciencias Med., claims that—

1. Quebracho diminishes the frequency of the respirations and cardiac contractions.
2. It strengthens and regulates cardiac contractions, either directly or through the nervous system.
3. This action is evident and immediate.
4. It has a manifest anti-dyspnœic action.

5. In nervous dyspnœas it must be tried in a greater number of cases.

6. It produces the same effect in dyspnœa from acute pulmonary affections.

7. Its prolonged administration produces no alteration in other organs or functions.—*Gaillard's Med. Jour.*

**Climate of Upper Georgia.**—The following remarks made at the Society of the County of Kings, Brooklyn, by Dr. P. R. Cortelyou, of Marietta, Ga., will doubtless be read with interest by many who are interested in the question of climate in the treatment of lung affections. What he says of Marietta will apply to Atlanta, and to the entire freestone region of Upper Georgia:

"While we all know that climate is, in no sense, absolutely curative of any disease, yet we all are aware that it exercises a very beneficial infl1uence in the treatment of many diseases, and especially to pulmonary and throat troubles. It was my misfortune a number of years ago, as some of the gentlemen present know, to be troubled with pulmonary difficulty, and I suffered from very severe hemorrhages, and was generally considered in a precarious condition, so much so that it was necessary for me to go away from this climate, and to find some place where there could be some chance of prolonging my days. My first experience was in Southern Georgia. I went to the South in 1875, to Thomasville, which is situated in the southwest of Georgia, about twenty-five miles from Florida, in a sandy section, the woods being pine, and the elevation being about six hundred feet above the level of the sea. I passed the winter there and found the climate, in many respects, very delightful and very beneficial to me during the winter. The average temperature for that section for the winter is about 65°. The weather is such, of course, as to allow one to be in the open air a great deal of the time. The main difficulty experienced by myself, and the same difficulty I found was experienced by others, was in the dampness. There was a good deal of moisture and considerable foggy weather, which affected many very unpleasantly, many others more than myself, and also in the feeling, especially towards the spring, of relaxation and debility. I found that in the morning I did not feel as refreshed after sleep as I desired. I then came north in the spring, and after remaining in Brooklyn for about two months I was troubled again with my old difficulty, hemorrhages and general bad health. I then went back to the southern portion of Georgia, and found that as I had done so badly in coming north in summer, I would remain in the Southern part of Georgia during the summer. I remained there until about the middle of July, by which time I got enough of summer. The result was typhoid fever, before the summer was finished. I made up my mind, therefore, that however the climate might agree with me in winter, it would not do for me in summer. My idea, then, was to find a climate suitable all the year round; and that, it seemed to me after careful study, was the climate which I desired. This changing from one place to another, I found, caused me to lose about as much as I gained, and if I could find a climate where I could live throughout the year, I could gain ground. I then went to upper Georgia, and finally located at Marietta, situated in lat. 34°, about twenty miles north of Atlanta, with an elevation of about one thousand one hundred and fifty feet above the level of the sea. The country is rolling, and the soil is of a red chocolate color, similar to that which prevails in eastern Tennessee and Virginia. The water is "free-stone," but there is an absolute freedom from all malarial troubles so prevalent in many of our Southern States. Especially in Florida, and sometimes Georgia, I found the atmosphere was more exhilarating and bracing. The average temperature for the winter months is 50°, and for the summer 75°; but I may say that during my residence there I have suffered less from heat during summer than I used to while a resident of Brooklyn. The temperature at 90° does not affect me there as unpleasantly as a temperature of 80° here. I presume one reason for this is freedom from moisture, and the dryness of the atmosphere. The amount of rain fall during the year averages, I think, about forty-five inches. I am not certain in regard to these figures, however, as it is sometime since I looked them up: I simply repeat them as they happen to come to my mind.

When I first went to Marietta, I was suffering from debility, unable to attend to any business, scarcely able to get around; but I found my residence there every month seemed to improve me; and for the past two or three years I have considered myself as certainly not on the invalid corps, and have endeavored to do what I could in the way of practice in that section. I have been thrown in contact with a good many persons who have gone South for pulmonary troubles, who have visited various sections of country, not only in the far South, but California and Texas, and their experience has been, many of them, very similar to my own; that while we find that other climates may be more equable in temperature, still, in the long run, invalids do not do so well as they do in the section of Upper Georgia, of which we are speaking. I think, of course, you are all aware that one reason why persons derive so little benefit from a change of climate is that they leave the matter too long before seeking a change—they seek it as a last resort. Many patients have gone to Marietta whom I have been obliged to send home again so that they may die among their friends. They went there too late, and only as a last resort. A number of cases have come under my observation—where persons have gone early and remained there, who have been very much benefitted permanently. Again, another trouble with persons who leave home for another climate on account of pulmonary trouble, is that they depend too much upon the climate. They go there and sit down, thinking all they have to do is to breathe in the air and breathe in health, and they make no effort to invigorate their systems by exercise and proper hygiene and diet. And then others, of course, are troubled with home-sickness; having nothing to occupy their minds they begin to worry, and as soon as the weather gets a little warm they are afraid they are going to be injured by a residence in a hot Southern climate, and, therefore, they feel that they must go home. Consequently they come North, probably reaching here in the midst of a snow storm, or in a cold eastern storm. And, as has been the case with some patients whom I have treated, they return to New York in good condition, but in a few months take pneumonia and die.

I think of nothing further interesting to the members to be said at the present time. However, I am very well assured that in my own case this removal has certainly been the best thing that I could have done, as it has been in the case of others with whom I am acquainted. Of course, as I said before, there is no one climate that will suit every case. There is no climate under heaven that will cure every case. Tuberculosis carries off a vast number of the human race, and we have no panacea for it. I am satisfied that with a reasonably fair climate, together with other measures, there is a reasonable hope for benefit; and I think the great mistake that many, aud I may say the majority of patients make, is that as soon as they feel benefitted, and as I said before, as soon as the spring comes, they want to return home. They are not willing to make the place which agrees with them a permanent residence. In my opinion every one who goes to a new climate for the purpose of regaining health, and finds that he is benefitted, should remain one year, at least, after all signs of trouble have disappeared; but it is difficult to make people believe this. Now I have in my mind a gentleman from Boston who had been in the habit of going South for ten years past. He spends the greater portion of the winter at Marietta, and does extremely well; but he comes North in the summer, and very often has a set back which puts him about where he was the previous fall. If he had remained there during the entire season for the past five or six years, I have no doubt but that he would have been entirely relieved of his trouble. I have endeavored to get him in that way of thinking, and he has decided to remain there this coming summer. Very many think that because the winters are mild the summers are exceedingly long and hot. The summers are really exceedingly pleasant. We rarely have a temperature above ninety degrees in the shade; and the nights are, also, very comfortable; and I don't think there were half a dozen nights during the past summer when I was kept awake on account of the heat, or when I did not need some covering. Another advantage of that section—I speak especially of Marietta, but many sections in upper Georgia are fully as favorable as to climate; the conditions are all the same, but we have a little more elevation than the other sections—is the absolute freedom from malarial troubles. And further, and this is the point to which I wish to direct your attention, we are easy of access. I think that is a decided benefit in many cases. I hear a good deal said about New Mexico. Well, when an invalid attempts to go there, away from his friends, he has a very tiresome and tedious journey, and the facilities for comfortable living there are, in many parts of the country, difficult to procure, so that if one requires any special luxuries it is difficult to get them. At Marietta we are situated conveniently to Atlanta, where we can get anything that can be had here; and persons located there are certainly very easily reached by friends, if necessary; and so they feel that they are not entirely isolated from their friends and families as they are when they go to New Mexico or even California."

**Enlarged Tonsils.**—Dr. Chisolm, in a paper read before Baltimore Academy of Medicine, says: "The tonsil is a very spongy gland full of holes. It is made up of infoldings of mucous membrane, by which crypts, follicles, or flask-shaped cavities are formed, with outlets upon the inner surface of the throat. In viewing the hypertrophied tonsil, small round depressions seem to honeycomb the surface. As many as fifteen or twenty can be counted. A probe will enter these open mouths without impediment, and will sink down into the innermost depths of the gland. These convolutions, if they may be so called, increase immensely the surface of the tonsil. Such crypts are lined with lymphoid follicles and epithelial cells. This lining membrane is much more delicate and destructible than that covering the exposed faucial surface of the gland.

In chronically enlarged tonsils, whether due to hypertrophy of the stroma or connective tissue of the organ, or to enlargement of the follicular structure, the epithelial elements of the follicles, the crypts and their outlets are not obliterated. In follicular hypertrophy the enlarged gland seems irregularly nodulated. The open mouths of the follicles are also enlarged and rendered conspicuous by tenacious exuding secretions. When the connective tissue elements are increased, the swelling would be firmer and more smoothly rounded, with shrunken follicles, but they still exist, although much contracted.

In the distribution of sensitive nerves, the exposed surfaces receive the larger supply according to rule, and the interior surfaces of the follicles are to a certain extent deficient in common sensation. Here, then, we have in these deep pits a much more extended, less sensitive and more easily influenced surface, to which destructive agents can be readily applied without annoying the throat proper. Caustics, if buried in the substance of the tonsil, will soon give evidence of the much desired shrinkage.

Among the various caustics for local use in causing shrinkage of tonsillar hypertrophies, I have found the chloride of zinc the most available and the least annoying to the patient. I employ it in the following manner: A piece of wire, the size of a fine knitting needle, is roughened for a half inch from one end, so that it may hold firmly a fibre of absorbent cotton twisted upon it by rotation between the fingers. This cotton wrapping, when dipped into a saturated solution of chloride of zinc, will be ready for use. This medicated cotton-lined probe is thrust to the very bottom of a crypt, and kept there several seconds. When it is withdrawn, the whitened orifice alone marks to the eye the cauterization. By renewing the cotton for each follicle, several may be thoroughly cauterized at the same sitting, without causing any annoying irritation to the throat. A very few applications will cause the gland to shrink, as will be seen one week after the destructive cauterization has been made to the interior of the follicles. The more thorough the application, the greater the effect produced. I have tried chromic acid, but find it more diffusible than the zinc caustic. Although I used the chromic acid with care, it extended over a part of the surface of the tonsil, causing pain, and producing a soreness of the throat which was complained of for several days. The cotton is withdrawn upon the probe and is not left in the tonsil.

If several crypts are cauterized, it is readily seen how a much larger surface can be influenced, as is exhibited by the entire face of the tonsil, with the very marked advantage of causing no surface ulceration, and therefore avoiding the necessary accompaniment—the sore throat.

The suggestion had been made by Dr. F. Donaldson, to puncture the tonsil with a sharp instrument and insert a crystal of chromic acid into the little wound through the open mouths of the crypts leading down into bottle-shaped cavities. We have a much more convenient access to the interior of the gland, and the caustic application is in every way a bloodless one. In attempts at destroying an hypertrophied tonsil by caustics, the destructive agent used should clearly be inserted within these blind chambers, and should not be applied for the excoriation of the side of the throat."

**Syphilitic Test.**—Dr. H. C. Wood (Med. Age.) prescribes the following test for syphilis in doubtful cases: "If I find that ten grains of iodide of potassium three times a day produces symptoms of iodism I am almost sure that the case is not one of specific disease. If, on the other hand, the patient takes from one-half to one drachm and waxes fat thereon, I am almost sure that he is the subject of specific disease."

**Chloroform Albuminuria.**—In man, after the administration of chloroform vapor, as in a prolonged operation, the urine sometimes contains albumen for a short period. Mr. Bouchard has recently read a paper on death following the subcutaneous injection of chloroform in animals. The injection of one cubic centimeter of chloroform, having a mean weight of seventeen hundred and nine grams, under the skin of the thigh of rabbits, was always followed by albuminuria and death in three days. If the dose injected be less, death may ensue after one or more injections. In the dog the injection of one cubic centimeter of chloroform per kilogram of body-weight gives rise neither to albuminuria nor death.—*Lancet.*

**Treatment of Fistula in Ano.**—Dr. Poingt claims (Le Courrier Medical) that any fistula amenable to treatment by the elastic ligature may be cured by simple drainage of the fistulous tract. The drainage-tube is to be inserted by means of a stylet passed up the tract from the external opening. At the end of two or three weeks the drainage-tube falls out, after having destroyed the superficial wall of the fistula. A granulating surface of small extent is left, which rapidly heals by cicatrization. The procedure is wholly painless, and the patient may pursue his ordinary avocations during the entire course of treatment. The operation is never followed by any of those serious complications sometimes seen after the cutting operation.—*Southern Clinic.*

